# Puumala Virus Infections Associated with Cardiovascular Causes of Death

**DOI:** 10.3201/eid1901.111587

**Published:** 2013-01

**Authors:** Anne-Marie Connolly-Andersen, Kristin Ahlm, Clas Ahlm, Jonas Klingström

**Affiliations:** Author affiliations: Umeå University, Umeå, Sweden (A.-M. Connolly-Andersen, K. Ahlm, C. Ahlm);; Swedish Institute for Communicable Disease Control, Solna, Sweden (J. Klingström);; Karolinska Institutet, Stockholm, Sweden (J. Klingström)

**Keywords:** Puumala virus, hemorrhagic fever with renal syndrome, HFRS, hantavirus, cause of death, cardiovascular diseases, emerging diseases, zoonosis, viruses

## Abstract

We studied the causes of death of patients in Sweden with diagnoses of hemorrhagic fever with renal syndrome (HFRS) during 1997–2009. Cardiovascular disorders were a common cause of death during acute-phase HFRS and were the cause of death for >50% of those who died during the first year after HFRS.

Hantaviruses cause 2 acute diseases: hemorrhagic fever with renal syndrome (HFRS) and hantavirus cardiopulmonary syndrome (HCPS). HFRS is caused by the prototypic hantavirus Hantaan and by Dobrava virus, Puumala virus (PUUV), and Seoul virus in Eurasia; HCPS is caused by Andes virus, Sin Nombre virus and related hantaviruses in the Americas. Case-fatality rates differ: <10% for HFRS and <40% for HCPS (1). PUUV causes HFRS in Europe; >225,000 cases of HFRS have been reported (2). One of the largest PUUV outbreaks recorded occurred in northern Sweden; an incidence of 313 cases per 100,000 persons was reported (3). The case-fatality rate for HFRS is 0.4% overall in Sweden and reaches 6% among elderly persons (4).

HFRS can cause pulmonary complications and HCPS can cause renal signs and symptoms, suggesting that these 2 diseases might have more in common than previously believed (5,6). However, as indicated by their respective names, HFRS is mainly considered a hemorrhagic fever with affected renal functions, and HCPS is characterized by severe cardiac and respiratory signs and symptoms (1). The primary causes of death during HCPS are known to be associated with cardiopulmonary failure (7). However, less is known regarding causes of death during the acute phase of HFRS and those that occur after HFRS related to possible sequelae of the illness. To explore patterns of death among persons who died during and after HFRS, we reviewed all causes of death of persons infected with PUUV in Sweden during 1997–2009.

## The Study

PUUV infection is a notifiable disease in Sweden, according to the Swedish Communicable Disease Act. Diagnosed cases are reported with each patient’s unique personal identity number to the Swedish Institute for Communicable Disease Control. These notifications are stored in a database; we obtained a permit from the Regional Ethical Review Board, Stockholm, to further analyze these patients. During the study period, HFRS associated with PUUV was diagnosed in 5,903 persons in Sweden. Of those, 59 persons lacked personal identity numbers, resulting in a database of 5,844 records (Table) that were compared to the Swedish cause of death registry administered by the Swedish National Board of Health and Welfare. The resulting study group, the Cause of Death (COD) cohort, comprised 238 deceased persons in whom HFRS had been diagnosed during 1997–2009 (median age at death 75 years, range 18–100, interquartile range [IQR] 66–82); 163 were male (median age at death 74 years, IQR 65–82), and 75 were female (median age at death 76 years, IQR 66–84).

**Table:** Characteristics of patients with diagnoses of HFRS, Sweden, 1997–2009*

Cohort	No. patients	No. (%) patients by sex			No. (%) patients by age group, y		
		F	M		Median age†	Age range	Interquartile range
HFRS	5,844	2,349 (40.2)	3,495 (59.8)		52	2–95	39–62
COD	238	75 (31.5)	163 (68.5)		71‡	18–94	62–78
HFRS, no PIN	59§	21 (37.5)	35 (62.5)		45‡¶	10–75	28–54

*HFRS, hemorrhagic fever with renal syndrome; COD, cause of death; PIN, personal identification number. Data from the HFRS database (Swedish Institute for Infectious Disease Control) were merged with data from the Cause of Death Register, National Board of Health and Welfare, identifying persons by PINs culminating in the cause of death (COD) cohort.  †The age in years of the patients at HFRS diagnosis according to the HFRS database. ‡Information regarding the age of 6 patients was missing. §There was a significant difference in ages of the persons in the COD cohort and entire HFRS cohort, and between those patients lacking PIN and the entire HFRS cohort (p<0.001 and p = 0.001, respectively, independent samples, Mann-Whitney U-test). ¶Information regarding the sex of 3 patients was missing. #The ages of persons in the “HFRS, no PIN” group were significantly younger than those in the COD cohort (p<0.001, independent samples, Mann-Whitney U-test).

**Figure 1:**
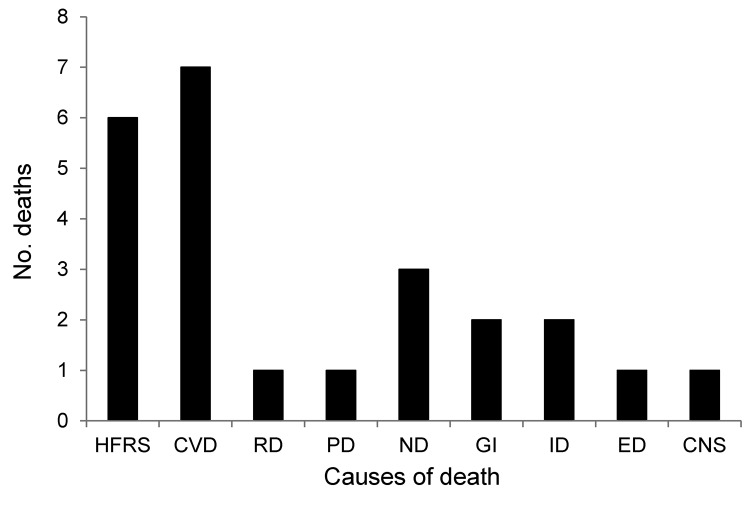
Main causes of death for patients in the acute phase of hemorrhagic fever with renal syndrome (HFRS), Sweden 1997–2009. Acute phase includes any death within 90 days of HFRS diagnosis. Data from the HFRS database, Swedish Institute for Communicable Disease Control, Cause of Death Register, National Board of Health and Welfare. HFRS, hemorrhagic fever with renal syndrome; CVD, cardiovascular disease; RD, renal disease; PD, pulmonary disease; ND, neoplastic disease; GI, gastrointestinal disease; ID, infectious disease; ED, endocrine disease; CNS, central nervous system disease.

Of the COD cohort, 24 (10%) patients (9 female, 15 male) died during the acute phase of HFRS (deaths within 90 d of HFRS diagnosis). The main cause of death for patients in the acute phase was categorized by using International Classification of Disease, 10th Revision (ICD-10), codes as specified by the World Health Organization: HFRS (A94, A98.5, N15.0); cardiovascular diseases (CVD) (I00–I99); renal diseases (N00–N39, excluding N15.0); pulmonary diseases (J00–J99, R06.8); neoplasms (C00–D48); gastrointestinal diseases (K55–K63); infectious diseases (A00–B99, excluding A94.0 and A98.5); endocrine diseases (E00–E99); and central nervous system (CNS) diseases (F00–F99, G00–G99). Unexpectedly, we found at least as many deaths caused by CVDs as by HFRS and related symptoms during the acute phase of HFRS (Figure 1). Causes of death for remaining persons in the cohort were neoplasms, infectious diseases, endocrine diseases, and CNS diseases (Figure 1). The mean ± SD time from diagnosis to death for persons in the COD cohort by disease was as follows: HFRS, 10.8 ± 5.3 d (n = 6); CVDs, 31.2 ± 14.4 d (n = 7); pulmonary, 15 d (n = 1); renal, 17 d (n = 1); neoplasms, 34.3 ± 20.1 d (n = 3); gastrointestinal, 44 ± 39.5 d (n = 2); infections, 77.5 ± 16.3 d (n = 2); endocrine, 22 d (n = 1); and CNS, 88 d (n = 1).

**Figure 2:**
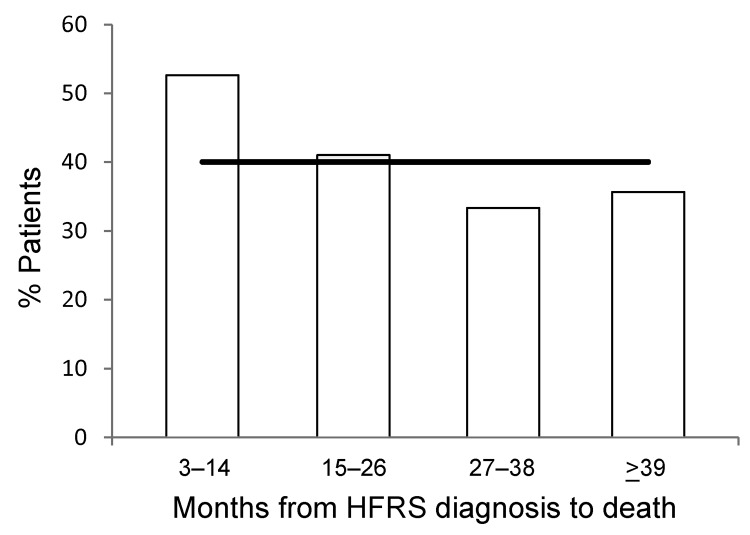
Main cardiovascular causes of death after the acute phase of hemorrhagic fever with renal syndrome (HFRS), Sweden 1997–2009. Bars indicate percentage of patients with cardiovascular diseases during 12-month intervals after HFRS as a main cause of death is displayed; horizontal line indicates the background rate of cardiovascular disease (40%) as main cause of death for the general population in Sweden. Data from the HFRS database, Swedish Institute for Communicable Disease Control, Cause of Death Register, National Board of Health and Welfare.

The prominence of CVD as a main cause of death for acute HFRS prompted us to explore patterns of cardiovascular-related deaths in the remaining decedents (n = 214, 90% of the COD cohort). We compared the percentage of cardiovascular-associated deaths by 12-month intervals according to when, after HFRS diagnosis, the patients died (Figure 2). During year 1 (months 3–14 after HFRS diagnosis), CVD deaths/total n = 10/19, median age at death 72 years (IQR 59–79); during year 2 (months 15–26), n = 16/39, 72 years (62–81); and during year 3 (months 27–38), n = 9/27, 73 years (66–81); and year 4 (>39 months), n = 46/129, 77 years (68–84). A disproportionate percentage of deaths were caused by CVDs for those who died during the first 12 months after acute HFRS compared with later and with deaths among the general population of Sweden (Figure 2). The proportion of CVD as cause of death during the second 12-month interval and later after diagnosis of acute HFRS was similar to that for the general Swedish population (Figure 2), indicating that HFRS might cause a transient elevated risk for death caused by CVDs. We found a significant difference (p<0.001) between the ages of the deceased persons and those of all persons with HFRS (Table), suggesting that age could have a confounding influence on the results of this study.

## Conclusions

Our finding indicate that CVD is a common cause of death from acute HFRS and might also be overrepresented as cause of death in the year after the acute phase of HFRS. PUUV infection is most likely a trigger of cardiovascular events that eventually lead to death rather than directly causing CVD, which can take 1–2 decades to develop. Other infections have been shown to trigger acute cardiovascular events (8) (e.g., compared with noninfected persons, persons with respiratory viral infections have 2–3× increased risk for acute coronary syndromes soon after infection) (9). The elevated risk is more pronounced during the acute phase of respiratory viral infection but can still be increased 3 months afterwards (9). Abnormal electrocardiograms (10,11) and myocarditis (12) have been observed during the acute phase of PUUV infection, indicating an association between PUUV infection and cardiovascular disorders. Furthermore, PUUV antigen has been found in cardiac tissue (6), and other studies have documented the essential role of coagulopathy in HFRS disease severity (13–15). Moreover, >25% of HFRS patients in Sweden had evidence of disseminated intravascular coagulation, which correlated with HFRS disease severity (14). Taken together, data from these studies (6,10–15) and the present data indicate a possible association between PUUV infection and precipitation of CVD events that could eventually lead to death.

A weakness of this study is the small sample number. Furthermore, this study is based entirely on data from public health registries, which poses the limitation of the inability to validate the cause of death data, including the risk for coding errors, which could affect the overall interpretations of our results. 

Our data suggest it would be prudent for health care providers to monitor elderly patients for affected cardiovascular functions during the first year after a diagnosis of HFRS. This potential association between CVDs and HFRS deserves further consideration in other cohorts of persons in whom HFRS was diagnosed, particularly in countries where more severe hantaviruses, such as Hantaan and Dobrava viruses, exist and in countries with large numbers of HFRS cases.
